# Development and Biological Properties of a New Live Attenuated Mumps Vaccine Strain

**DOI:** 10.3390/vaccines13080879

**Published:** 2025-08-20

**Authors:** Xue Song, Xiumei Ren, Yang Song, Shengbao Yang, Kailang Lu, Yan Zhang, Jiankai Liu

**Affiliations:** Beijing Minhai Biotechnology Co., Ltd., Beijing 102609, China; songxue@biominhai.com (X.S.);

**Keywords:** mumps virus-365, measles–mumps–rubella vaccine, immunogenicity, safety

## Abstract

**Background/Objectives**: This study aimed to develop a new attenuated live mumps vaccine strain and determine its biological properties and effectiveness. **Methods**: Plaque purification and amplification were performed in chicken embryo cells. Candidate live attenuated mumps MuV-365 strain sequencing was performed. After evaluating the potential neurotoxicity of the MuV-365 mumps strain, a preclinical safety evaluation of measles–mumps–rubella (MMR) live attenuated vaccine containing the MuV-365 strain was performed to support the registration and application of the MMR vaccine. Finally, mumps neutralization antibody titers and the concentration of anti-serum mumps-specific IgG were determined to evaluate the immunogenicity and efficacy of the MuV-365 strain and MMR vaccine in mice and rhesus monkeys. **Results**: The plaque of the PL-KUM main seed virus was screened, and strains whose sequences were highly homologous to RIT4385 (JL-5 derived) were selected to amplify. The candidate live attenuated mumps MuV-365 strain was then developed. Safety evaluation results indicated that the MuV-365 strain had no potential neurotoxicity, and the MMR vaccine containing the MuV-365 strain also showed no significant safety hazard. The immunogenicity of MuV-365 strain in BALB/c mice was not inferior to S79 and PL-KUM. After two doses of the MuV-365 strain, the concentration of anti-serum mumps-specific IgG of the MuV-365 strain was significantly higher than that of the S79 strain (*p* < 0.01). In rhesus monkeys, the MMR vaccine had good immunogenicity against measles and rubella after one dose, while immunogenicity against mumps improved after two doses. **Conclusions**: The developed MuV-365 strain was genetically stable, with adequate safety and immunogenicity.

## 1. Introduction

Mumps, caused by the mumps virus (MuV), remains a serious threat to the health of children and teenagers. In most mumps cases, the clinical symptoms are parotitis, myalgia, malaise, fever, and headache. As a neurotropic virus, MuV infection also causes cerebrospinal fluid pleocytosis in one-third of patients and meningitis in 10% of patients. In very few cases, orchitis and oophoritis can also occur [1]. MuV, a member of the genus Rubulavirus within the family Paramyxoviridae, contains a single-stranded negative-sense RNA composed of 15,384 nucleotides [2]. The genome of MuV encodes six structural proteins (nucleoprotein, phosphoprotein, matrix protein, fusion protein, hemagglutinin–neuraminidase protein, and large protein) and three non-structural proteins (V protein, I protein, and small hydrophobic (SH) protein). MuV is divided into 12 genotypes (A-N, excluding E and M) according to the sequence of SH genes, which are distinct from each other [3], but MuV has only one serum type, so a mumps vaccine could theoretically protect against viral infections from all MuV genotypes.

Thanks to the development of mumps vaccines and routine vaccination, the global incidence of mumps infections has significantly decreased. In China, the predominant MuV is genotype F [4,5,6]. Researchers isolated a total of 451 strains of MuV between 2001 and 2007, of which 97.33% were identified as genotype F [4,5,7]. In 2008, China implemented the Expanded Programme on Immunization in response to the proposal of the World Health Organization in 1974 and introduced new vaccines, such as the hepatitis A vaccine and measles–mumps–rubella (MMR) vaccine, but there are still outbreaks of mumps infections among the vaccinated population in China every year [8]. Several studies have therefore confirmed that it is necessary to develop a more effective mumps vaccine against wild-type MuVs in China [9,10,11,12,13,14]. Some studies have focused on vaccines containing wild-type genotype F, but their effectiveness is limited compared to other vaccine strains, possibly because of genotype-specific antigenic differences between the vaccine and wild-type MuV strains [14].

IMUNA PHARM, A.S. (Slovakia), obtained the original strain Mumpsvax NC7713 Jeryl Lynn mumps vaccine strain for commercial mumps vaccine production from Merck in 1981. The master seed batch of PAVIVAC vaccine (batch number: 150182/3 PL-KUM) was prepared by passaging the Mumpsvax NC7713 Jeryl Lynn mumps vaccine strain three times at 34 °C on primary dog kidney cells. This batch was used to produce the PAVIVAC vaccine, which has been used in Slovakia and has shown good immune protection. In this study, we developed a new live attenuated mumps vaccine strain, MuV-365, based on 150182/3 PL-KUM. We then focused on investigating the preclinical safety of MuV-365-containing MMR and its neutralization capacity of one- and two-dose virus-induced antibodies against genotypes A, F and G.

## 2. Materials and Methods

### 2.1. Reagents and Kits

Newborn calf serum (NBS, #22011–8615) was purchased from Zhejiang Tianhang Biotechnology (Huzhou, China). We purchased 199 medium (#1500–030) from Yixing Saier Biotechnology (Yixing, China). Human blood albumin (#201910133A) was purchased from Grand Shuyang Life Sciences (Chengdu, China). Hydrolyzed milk protein was purchased from ThermoFisher Scientific (Waltham, MA, USA).

### 2.2. Cell Culture

Specific pathogen free (SPF) chicken embryos were purchased from Beijing Boehringer Ingelheim Viton Biotechnology (Beijing, China). After incubation for 9–11 days, chicken embryos with clear blood vessels were selected to prepare embryo cells. Chicken embryos were cut and digested, and the cell density of each chicken embryo obtained was (5–12) × 10^5^/mL. The chicken embryo cells were cultured with hydrolyzed milk protein solution supplemented with 2.5% NBS. Vero cells were obtained from the National Institute for Food and Drug Control and cultured with 199 medium supplemented with 10% NBS.

### 2.3. Mumps Virus Strains

150182/3 PL-KUM, which was prepared by passaging the Mumpsvax NC7713 Jeryl Lynn mumps vaccine strain and used to produce the PAVIVAC vaccine, was obtained from IMUNA PHARM, A.S. The mumps virus S79 strain was prepared by passaging from the National Reference of Live Attenuated Mumps Vaccine (non-human use; lot number 310018–200701). The main mumps vaccine strain in China is S79, which was derived from the Jeryl Lynn strain. The reference materials of the wild genotype A and G mumps virus were obtained from the Chinese Center for Disease Control and Prevention and prepared by passaging on Vero cells in Beijing Minhai Biotechnology Co., Ltd. (Beijing, China). Genotype F mumps virus was isolated and passaged on Vero cells by the Beijing Minhai Biotechnology Co., Ltd.

### 2.4. Animals

Forty CD-1 mice (20 mice/sex, 6–7 weeks old, male mice weighing 32.4–37.1 g, and female mice weighing 25.1–29.0 g) were purchased from Beijing Vital River Laboratory Animal Technology Co., Ltd. (Beijing, China) (SCXK (Beijing) 2016–0006). One hundred and twenty Sprague Dawley (SD) rats were purchased from Beijing Vital River Laboratory Animal Technology (SCXK (Beijing) 2016–0006). Six male New Zealand rabbits (2–5 months old, weighing 2.96–3.66 kg) were purchased from Suzhou Huqiao Biotechnology (SCXK (Su) 2020–0002). Forty-five female guinea pigs (5–6 weeks old, weighing 293–365 g) were purchased from Beijing Vital River Laboratory Animal Technology (SCXK (Beijing) 2016–0011). The animals were kept in a room at a temperature of 20–26 °C and humidity of 40–70%, with a 12 h day and night cycle. All animal experiments were reviewed and approved by the Ethics Committee of JOINN Laboratories (Suzhou). To protect animals used for scientific purposes, all animal experiment procedures conformed to the 8th Edition of the Institute of Laboratory Animal Resources Commission on Life Science, National Research Council.

### 2.5. Purification of the Candidate Live Attenuated Mumps MuV-365 Strain

#### 2.5.1. Plaque Purification and Amplification

Chicken embryo cells were separately seeded in six-well plates at a density of (1.8–4.2) × 10^6^ per well and then incubated at 37 °C in 5% CO_2_ for 2–3 days. The 150182/3 PL-KUM master seed (PL-KUM strain) was then serially diluted to 30 CCID_50_/mL and added to the six-well plates (1 mL/well) to infect the chicken embryo cells at 34 °C in 5% CO_2_. After co-culture for 1.5 h, the inoculum was removed, and the cell monolayer was overlaid with plaque medium prepared from 199 medium supplemented with 2% NBS and 1.5% agarose. This culture was incubated at 34 °C in 5% CO_2_, while monitoring the appearance of plaques.

Pipette tips (1 mL) were used to select the typical mumps virus plaques. A total of 440 plaques (passage 1) were selected, named MuV-1 to MuV-440; co-cultured with chicken embryo cells in six-well plates; and then incubated at 34 °C in 5% CO_2_ for 5–7 days. The virus solution (passage 2) was harvested when the cytopathy reached 75%. We selected a total of 69 strains with virus titers no less than 6.0 lgCCID_50_/mL for sequencing and sequence alignment. Working seed (passage 6) was constructed by co-culturing the virus solution with chicken embryo cells from generation to generation, and it was used to manufacture the mumps vaccine.

#### 2.5.2. The Plaque Shape in Vero and Chicken Embryo Cells

Vero cells were seeded in six-well plates at a density of 7.5 × 10^5^ per well, and chicken embryo cells were seeded in six-well plates at a density of 1.8–4.2 × 10^6^ per well. These were then incubated at 37 °C in 5% CO_2_ for 2–3 days. The selected mumps virus MuV-365 and PL-KUM strains were serially diluted in 199 medium to 50 CCID_50_/mL and added to the six-well plates (1 mL/well) to separately infect the Vero and chicken embryo cells at 34 °C in 5% CO_2_. After co-culture for 1.5 h, the inoculum was removed, and the cell monolayer was overlaid with plaque medium prepared from 199 medium supplemented with 2% NBS and 1.5% agarose. The culture was incubated at 34 °C in 5% CO_2_ for 7 days, while monitoring the appearance of plaques.

Upon confirmation of visible plaques, the agarose was removed, and 0.25% Coomassie Brilliant Blue staining solution containing 45% ethanol and 10% acetic acid was added to the six-well plates (1 mL/well). After staining for 15 min at room temperature, the six-well plates were washed with tap water, and the morphology of the plaque was observed and photographed.

#### 2.5.3. Amplification Curve of the MuV-365 Strain

The PL-KUM and MuV-365 strains were separately inoculated with Vero cells and chicken embryo cells at a multiplicity of infection (MOI) of 0.0006. A total of 10 mL of cell suspension containing virus was added to each T25 cell culture flask and incubated at 34 °C for 7 days. Three flasks were harvested in each group for virus titration, every day. Virus titration was conducted using a 50% cell culture infection dose assay in Vero cells.

### 2.6. Mumps Virus Sequencing

The total RNA of the mumps virus was extracted using a QIAamp MinElute Virus Spin Kit (Qiagen, Hilden, Germany), and the isolated virus RNA was reverse-transcribed into cDNA using a Super Script III First-Strand kit (Invitrogen, Waltham, MA, USA). The mumps virus genes were amplified with the designed primers and then sequenced by Ion Torrent Platform next-generation sequencing. The entire sequences from the RIT4385 strain were retrieved from GenBank (FJ211584), and multiple nucleotide and amino acid sequence alignments were performed using MEGA software version 7.0 (http://www.megasoftware.net/, accessed on 20 November 2024).

### 2.7. Preclinical Safety Evaluation of the MMR Vaccine Containing the MuV-365 Strain

#### 2.7.1. Potential Neurotoxicity Evaluation of the MuV-365 Mumps Strain

To evaluate the potential neurovirulence of the main seed of the MuV-365 mumps strain, 14 quarantinable (mumps-neutralizing-antibody-negative) rhesus monkeys were randomly divided into two groups (half male and half female) according to body weight. For the test group (five males and five females), with the help of the stereotactic apparatus, 0.5 mL of the MuV-365 mumps strain with a virus titer of 5.625 lgCCID_50_/mL was injected into each side of the thalamus on day 1 (day of surgery), at an injection rate of 0.5 mL/10 min. For the negative control group (two males and two females), the same volume of normal saline was injected at the same injection rate. During the test, the clinical observation, body weight, and temperature of the rhesus monkeys were regularly tested. The serum neutralization antibody of the negative control group was measured on day 22 and day 32, and that of the test group was measured once on day 22. After the euthanasia of rhesus monkeys on day 22 and day 32, histopathological and microscopic examinations were conducted.

#### 2.7.2. Single-Dose Toxicity Test of MMR Vaccine Containing the MuV-365 Strain

To evaluate the possible toxicity of MMR after a single subcutaneous injection of CD-1 mice, and to determine the toxicity 14 days after the end of the administration period, a total of 40 CD-1 mice were randomly divided into two groups according to their body weights before administration (day 1 represents the first day of administration). Mice in group 1 were given sodium chloride injection as a negative control (0 dose/mouse), and mice in group 2 were given MMR (one dose/mouse). The skin of the nape of the neck of the mice was selected for subcutaneous injection, and the dosage was 0.5 mL/mouse in a single dose. During the test, clinical observation (including observation near the cage after administration and local observation after administration), body weight, and food intake of the mice were regularly conducted. At the end of the 14-day observation period (day 15), all mice were euthanized, and gross anatomical evaluations were performed which included observing whether there were abnormalities in the surface and pores of mice, as well as the skull, chest, abdomen, pelvic bones, and their contents. After the experiments, all mice were anesthetized with isoflurane inhalation and subjected to abdominal aortic bloodletting to perform animal euthanasia.

#### 2.7.3. Repetitive-Dose Toxicity Test of the MMR Vaccine Containing the MuV-365 Strain

To evaluate possible toxic reactions after repeated subcutaneous injections of MMR in Sprague Dawley (SD) rats, rats were injected once every 3 weeks for 6 consecutive weeks (a total of three times) to observe possible toxic reactions or delayed recovery from toxic reactions for 4 weeks after the last administration. A total of 120 SD rats (60 rats/sex) were randomly divided into six groups according to their weights before administration (day 1 represents the first day of administration). Groups 1, 2, and 3 were the main test groups for the toxicological study (15 rats/sex/group), and groups 4, 5, and 6 (5 rats/sex/group) were the satellite group for the immunological index study. Rats in groups 1 and 4 were given normal saline injections as negative controls (0 dose/rat and 1.5 mL/rat), rats in groups 2 and 5 were subcutaneously injected with MMR at a dose of 1 dose/rat (0.5 mL/rat), and rats in groups 3 and 6 were subcutaneously injected with MMR at a dose of three doses/rat (1.5 mL/rat). One dose corresponded to a single injection dose per person for human use (3.3 lgCCID_50_ of measles and rubella virus and 4.0 lgCCID_50_ of mumps virus). The skin of the nape of the neck of the rats was selected for subcutaneous injection, which was administered once every 3 weeks for 6 consecutive weeks, and was administered three times in total (on day 1, day 22, and day 43). During the test, clinical observations (including local observation after administration), body weight, food intake, body temperature, eye examination, blood count, coagulation function, blood biochemistry, urine analysis, T-lymphocyte subsets (CD3+, CD4+, CD8+, or CD4+/CD8+), and cytokines (IL-2, IL-6, TNF-α, IFN-γ, or GM-CSF) were conducted. The first 10 rats/sex/group of the main experimental group were euthanized 3 days after the last dose (day 46), and the last 5 rats/group/sex were euthanized at the end of the 4-week recovery period (day 72). Then, we examined the organ weight, gross anatomy, and histopathology.

#### 2.7.4. Local Tolerance Test of the MMR Vaccine Containing the MuV-365 Strain

To observe the local stimulation response of MMR after a single subcutaneous injection in New Zealand rabbits, the local tolerance test was performed. A total of six male New Zealand rabbits were used in this experiment. Single-point injection of normal saline (0 dose/rabbit) was performed on the left nape of the New Zealand rabbits as the negative control area, and a single-point injection of MMR vaccine (1 dose/rabbit) was performed on the right nape of the New Zealand rabbits as the experimental area. The injection volume was 0.5 mL per rabbit. On day 4 (day 1 represents the day of the subcutaneous injection) after subcutaneous injection, three rabbits were randomly selected for euthanasia, and the skin and subcutaneous connective tissue at the administration site were collected for histopathological examination. On day 15 after subcutaneous injection, the same procedures were performed on the remaining three rabbits. To perform animal euthanasia, all New Zealand rabbits were anesthetized with intramuscular injections of ketamine (30 mg/kg) and xylazine (5 mg/kg). When the corneal reflex of rabbits had disappeared, carotid/femoral artery bloodletting was performed.

#### 2.7.5. Systemic Anaphylaxis Using the MMR Vaccine Containing the MuV-365 Strain

Guinea pigs were sensitized by subcutaneous injection of MMR vaccine three times on day 1, day 3, and day 5, and were stimulated with MMR vaccine intravenously on day 19 and day 26 to observe whether the guinea pigs had rapid allergic reactions. Forty-five female Hartley guinea pigs were randomly divided into five groups of nine per group according to their weights before administration (day 1, the first day of administration). The negative control group (group 1) was given a sodium chloride injection; the positive control group (group 2) was given human blood albumin; the low-dose (group 3) and high-dose (group 4) groups were given MMR; and the human blood albumin and gelatin-free control groups (group 5) were sensitized by MMR vaccine and further stimulated by using semi-finished MMR. Sensitization was accomplished by subcutaneous injection of both hind limbs on day 1, day 3, and day 5. Fourteen days after the last sensitization with the MMR vaccine (day 19), the foot of the first three animals in each group was stimulated by intravenous injection. After stimulation, anaphylaxis occurred in the low- and high-dose groups of the test group, but no anaphylaxis occurred in the control group without human blood albumin and gelatin. The remaining animals in the low- and high-dose groups were therefore stimulated twice on the same day (day 19). One week later (day 26), the remaining animals of the negative, positive, and human blood albumin and gelatin-free groups were stimulated twice. The dosage and anaphylaxis capacity of animals in each group are shown in Table 1.

For the supplementary test, after the stimulation injection on day 19, animals in the test group showed symptoms of anaphylaxis. Two healthy guinea pigs that had not been used before were injected intravenously with semi-finished MMR in the high-dose test group to observe whether there were symptoms of anaphylaxis caused by the test subjects, and to provide a reference for further studies.

To perform animal euthanasia, all guinea pigs were anesthetized with CO_2_ inhalation, and then femoral artery bloodletting was performed.

### 2.8. Preclinical Immunogenicity Evaluation of the MuV-365 Strain and MMR Vaccine Containing the MuV-365 Strain

#### 2.8.1. Preclinical Immunogenicity Evaluation of the MuV-365 Strain in BALB/c Mice

Female BALB/c mice aged 4–6 weeks were purchased from the Beijing Fuhao Experimental Animal Breeding Center. Mice were immunized with four kinds of A genotype mumps virus (MuV-365, S79, PL-KUM, and wild A genotype), using the G and F genotypes, by intramuscular injection (i.m.) at 0.1 mL per mouse with 10^5^ CCID_50_ live virus, using 10 mice/group. Re-immunization was performed after 28 days of immunization, and blood was collected after 28 days (one dose) and 42 days (two doses) post-infection. All sera were analyzed to quantitatively and qualitatively detect mumps-specific IgG antibodies using the Serion ELISA classic MuV IgG test kit (Institut Virion\Serion GmbH, Wurzburg, Germany) and stored at −60 °C until use for neutralization tests.

#### 2.8.2. Preclinical Immunogenicity Evaluation of the MMR Vaccine Containing the MuV-365 Strain in Rhesus Monkeys

Blood samples from rhesus monkeys were collected before immunization, and the monkeys with negative antibodies, anti-measles virus, anti-mumps virus, and anti-rubella virus antibodies were screened. A total of 15 negative control monkeys (seven males and eight females) were used in the experiment. MMR was administered via subcutaneous injection at the lower border attachment of the lateral deltoid muscle of the upper arm. Each monkey was immunized once on day 1 and once on day 28, with an immunization dose of 1 dose/monkey each time. Clinical observations of the animals (including but not limited to death, mental state, behavioral activity, morbidity, respiration, secretions, feces, diet, and water intake, etc.) were performed daily during the trial period. Blood samples at 28 days post-infection (before secondary immunization), 42 days post-infection (dpi), and 56 days dpi were collected and stored at −60 °C until use for neutralization tests.

### 2.9. Neutralization Test

In the neutralization assays, stored serum samples underwent heat inactivation (56 °C, 30 min), followed by dilution in maintenance medium. A series of two-fold serum dilutions was prepared, and 50 μL aliquots of each dilution were dispensed into duplicate wells of a 96-well microplate. Subsequently, 50 μL of virus solution, standardized to 50 CCID_50_/50 μL, was introduced into each well. After thorough mixing, the plates were incubated for 1 h under 5% CO_2_. Following this incubation, 100 μL of Vero cell suspension was added per well. Appropriate viral and cellular control wells were established on the same plate. Plates were then returned to the 5% CO_2_ incubator, and cytopathic effects were monitored daily over 10 days. For this study, a neutralization titer ≥1:2 was designated as the positive threshold.

### 2.10. Statistical Analysis

Data were analyzed using a two-tailed Student’s *t*-test with Excel (Microsoft) and were expressed as the mean ± standard deviation (SD) of independent experiments. Values of *p* < 0.05 indicated statistically significant differences.

## 3. Results

### 3.1. Plaque Purification and Amplification of the MuV-365 Strain

**Figure 1:**
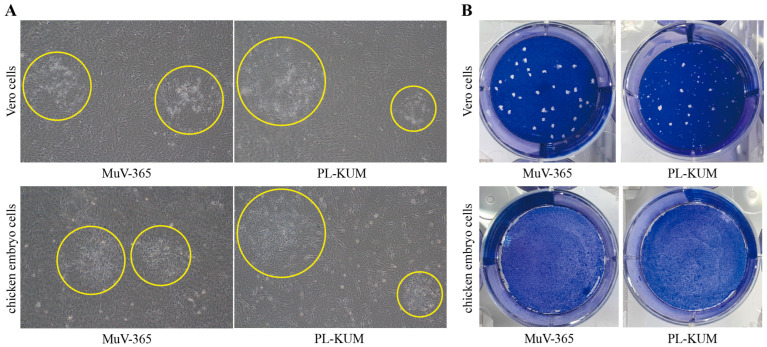
Viral plaque shape in Vero and chicken embryo cells. The microscopic observation of the PL-KUM strain and MuV-365 strain plaques in Vero cells and chicken embryo cells (**A**), the yellow circle indicates the virus plaque. The Coomassie Brilliant Blue staining of the PL-KUM strain virus and MuV-365 strain virus plaques in Vero cells and chicken embryo cells (**B**).

The morphology of the plaques is shown in Figure 1. The MuV-365 strain virus plaques were large, and the morphology of the viral plaques was uniform in both Vero and chicken embryo cells (Figure 1A). However, the size and morphology of the PL-KUM strain virus plaques were uneven in both Vero and chicken embryo cells. Similar results were observed after staining with Coomassie Brilliant Blue, which showed that the MuV-365 strain virus plaques were large and almost uniform in size, while the plaques of the PL-KUM strain virus had varying sizes and shapes (Figure 1B).

**Figure 2:**
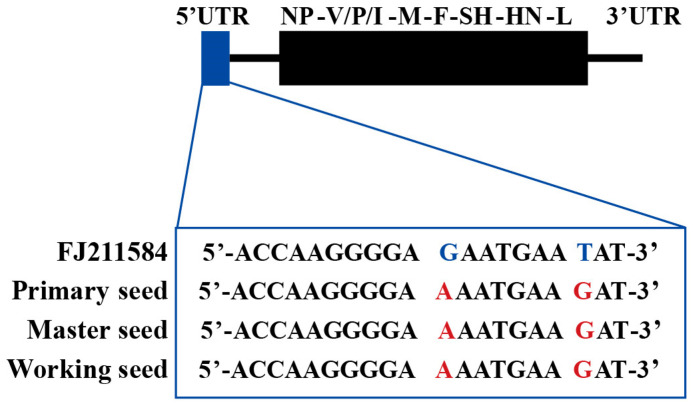
Sequencing analysis of MuV-365. The sequence of primary, master and working seed was identical to the RIT4385 strain with two mutations in positions 11 and 18 of 5′UTR.

A total of 440 monoclonal plaques were selected, using microscopy. The selected plaques were passaged to the second generation according to the cytopathic morphology (typical fusion), cytopathic progression, cytopathic degree (not less than 50%), and virus titer (not less than 6.0 lg CCID_50_/mL). Sixty-nine passage 2 strains were selected for full-length sequencing. The virus selected for passage had no sense mutations that could alter the amino acid sequence, except for two base mutations at the 5′-UTR, when compared to FJ211584. Among these, MuV-365 was selected to construct a working seed because of its typical cytopathic morphology and virus titer. The sequences of primary, master, and working seeds are shown in Figure 2.

### 3.2. Amplification Curve of the MuV-365 Strain

**Figure 3:**
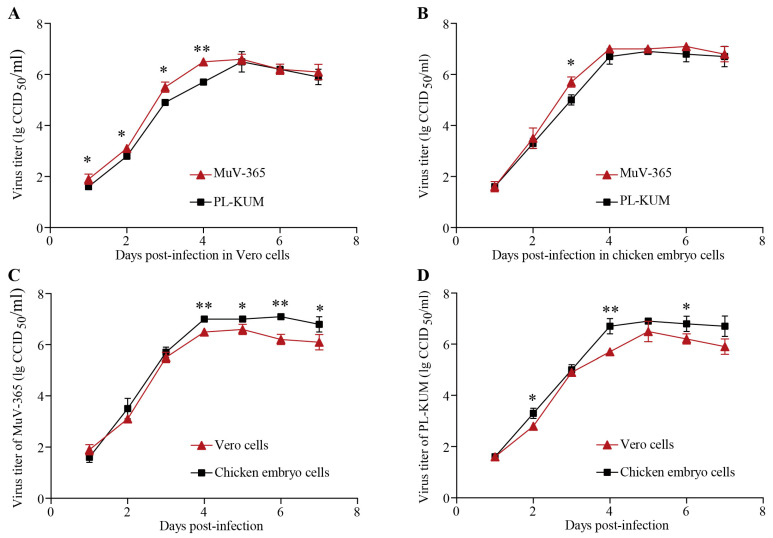
Amplification curve of PL-KUM and MuV-365 strains. The expansion rates of the PL-KUM strain and MuV-365 strain in Vero cells (**A**) and chicken embryo cells (**B**). The expansion rates of the MuV-365 strain (**C**) and PL-KUM strain (**D**) in different cells. Data are presented as the mean ± SD, *n* = 3; * *p* < 0.05 and ** *p* < 0.01 by unpaired *t*-test.

The PL-KUM strain and MuV-365 strain were inoculated with the same MOI into Vero and chicken embryo cells, respectively. As shown in Figure 3A,B, the MuV-365 strain had a faster expansion rate in Vero and chicken embryo cells, but there was no significant difference in virus titer during the plateau phase. As shown in Figure 3C,D, the MuV-365 strain and PL-KUM strain were both more suitable for proliferation in chicken embryo cells.

### 3.3. Preclinical Safety Evaluation of the MMR Vaccine Containing the MuV-365 Strain

#### 3.3.1. Potential Neurotoxicity of the MuV-365 Mumps Strain

No animal died during the experiments, and no symptom of nerve paralysis was observed. During the experiment, no change in body weight was observed. One animal in the test group had a transient increase in body temperature, which is a common adverse reaction after mumps vaccination. Other animals had normal temperatures. For the neutralizing-antibody assay, the serum mumps-neutralizing antibody of one animal in the negative control group was positive on day 22 and day 32, while in the test group, the positive conversion of the serum mumps-neutralizing antibody on day 22 was 90%. At the end of the experiment, no significant abnormality was found in the gross anatomy of all animals. Microscopically, needle stitch reactions were observed in both the negative control and the test groups, and in addition to needle stitch reactions, general reactions (intrinsic reactions) caused by the vaccine were also observed in the test group.

The results showed that in rhesus monkeys, all mumps-neutralizing antibodies were negative before inoculation, and the animals were mumps virus-susceptible. No animal death was observed within 48 h after injection, and the numbers of animals in the negative control group and the test group (histopathological examination showed chronic progressive inflammation at the thalamic stitch, and the injection site was confirmed as in the thalamus) were 3 and 10, respectively. After the thalamic injection on day 22, 90% of the animals in the test group produced an immune reaction. However, on day 32, one of the four control monkeys had neutralizing antibodies, suggesting that intra-thalamic injection may have caused lateral transmission. Transient fever was also observed in some animals after intra-thalamic inoculations. However, there were no obvious clinical symptoms of the central nervous system, or any other toxic side effects, except for slight inflammation in local brain tissue and vascular sheath formation in the test group.

#### 3.3.2. Single-Dose Toxicity Test of the MMR Vaccine Containing the MuV-365 Strain

During the experiment, no animal died or showed any signs of near death in each group. No abnormal clinical symptoms were observed in all groups of animals, and no abnormal changes were observed by naked-eye observations of the injection sites. Compared with the same-sex negative control group during the same period, there was no change in body weight, body weight gain, or food intake related to the test substance in the animal test group at different periods. In this experiment, the MMR was administered subcutaneously to CD-1 mice at a dose of one dose per mouse, with a maximum tolerated dose of one dose per mouse (0.5 mL/mouse).

#### 3.3.3. Repetitive-Dose Toxicity Test of the MMR Vaccine Containing the MuV-365 Strain

During the repetitive-dose toxicity test, no animal died or showed any sign of near death. No abnormal clinical symptom related to the test substance was observed, and no abnormal change was observed at the administration site in any of the groups of animals. Compared with the same-sex negative control group during the same period, no statistically significant change was observed in the body weight, body weight gain, food intake, body temperature, blood cell count, coagulation function, serum biochemical indicators, urine analysis, T lymphocyte subsets (CD3+, CD4+, CD8+, or CD4+/CD8+), cytokines (IL-2, IL-6, TNF-α, IFN-γ, or GM-CSF), organ weight, visceral to body ratio, and visceral-to-brain ratio.

Regarding the results of gross and histopathological examinations, mild infiltration of inflammatory cells in the dermis/subcutaneous tissue of the administration site was observed in 2/20 (1 female and 1 male) rats from the low-dose group and 5/20 (2 males and 3 females) from the high-dose group on day 46, which was believed to be related to the test substance in the low- and high-dose groups, respectively. However, the rats that experienced these symptoms showed complete recovery on day 72.

In summary, under the conditions of this experiment, SD rats were repeatedly subcutaneously injected with MMR at doses of one dose/rat and three doses/rat for 6 consecutive weeks (once every 3 weeks for a total of three doses). The No Observed Adverse Effect Level was three doses/rat. Local irritant reactions related to the test substance were observed at the administration site, but complete recovery was observed after 4 weeks.

#### 3.3.4. Local Tolerance Test of the MMR Vaccine Containing the MuV-365 Strain

During the experiments, no animal showed any signs of death or near death, and no abnormal clinical symptom related to the test substance was observed. Furthermore, no abnormal change was observed at the administration site. On day 4 and day 15, no change was observed in the administration site, using gross or microscopic evaluation. Light subcutaneous inflammatory cell infiltration was observed microscopically at the administration site of euthanized animals (left and right) on day 4. Because similar lesions were also noted in the negative control group and were considered to be unrelated to the sample. At the end of the recovery period (day 15), slight inflammatory cell infiltration was observed microscopically in the muscle layer of the right administration site. This lesion occurred in only one case, and the degree was mild. It was considered to be unrelated to the sample, and no obvious pathological change was observed in the remaining animals. In summary, MMR given to New Zealand rabbits by single subcutaneous injection at a dose of 1 dose/0.5 mL/vaccine showed no irritation at the administration site.

#### 3.3.5. Systemic Anaphylaxis from the MMR Vaccine Containing the MuV-365 Strain

During the experiment, no abnormal reaction related to administration was observed in all animals before stimulation. The clinical observations of animals in each group after day 19 of foot vein stimulation are shown in Table 2.

The clinical observations of animals in each group after day 26 foot vein stimulation are shown in Table 3.

The results of the supplementary tests showed no symptoms of anaphylaxis observed in the high-dose, intravenous injection group. Under the conditions of this experiment, the guinea pigs were sensitized by subcutaneous injection three times at doses of 0.1 dose and 1 dose of MMR (equivalent to 20 and 200 times the clinical intended dose, respectively), and stimulated intravenously at the double sensitization dose 14 days after the last sensitization. Guinea pigs were observed to have rapid allergic reactions.

Guinea pigs were sensitized by subcutaneous injections three times at a dose of 0.1 dose and 1 dose of MMR (equivalent to 20 and 200 times the clinically intended human dose, respectively), and stimulated intravenously at the double sensitization dose at 14 days and 21 days after the last sensitization (semi-finished MMR vaccine without human blood albumin and gelatin). No rapid onset of anaphylaxis in guinea pigs was observed.

### 3.4. Preclinical Immunogenicity Evaluation of the MuV-365 Strain and MMR Vaccine Containing the MuV-365 Strain

#### 3.4.1. Preclinical Immunogenicity Evaluation of the MuV-365 Strain in BALB/c Mice

**Figure 4:**
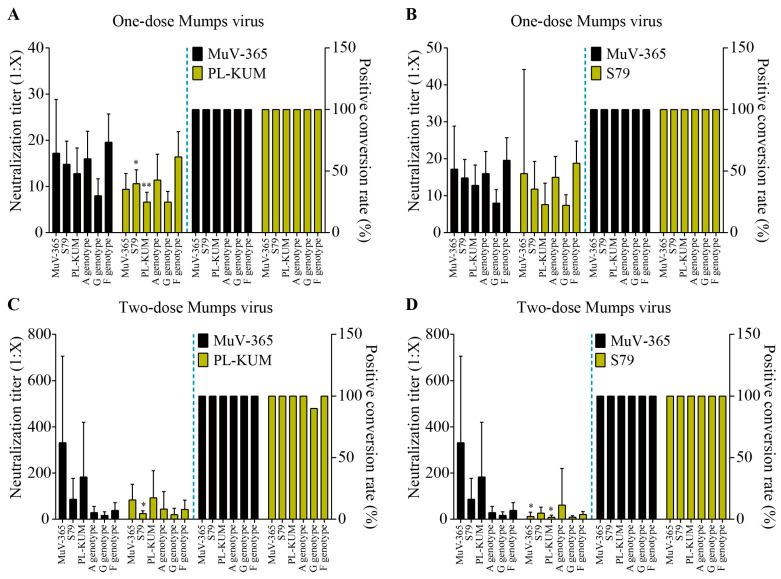
The mumps neutralization antibody titers. The mumps neutralization antibody titers against MuV-365, S79, PL-KUM, wild A, G, and F strains induced by one dose of MuV-365 and PL-KUM, respectively (**A**). The mumps neutralization antibody titers against six strains were induced by one dose of MuV-365 and S79, respectively (**B**). The mumps neutralization antibody titers against six strains were induced by two doses of MuV-365 and PL-KUM, respectively (**C**). The mumps neutralization antibody titers against six strains were induced by two doses of MuV-365 and S79, respectively (**D**). Data are presented as the mean ± SD, *n* = 10; * *p* < 0.05 and ** *p* < 0.01 by unpaired *t*-test.

The immune protection of MuV-365 strain antiserum in one dose against PL-KUM and S79 strains was significantly higher than that of the PL-KUM strain (*p* < 0.05 or *p* < 0.01). There was no significant difference in immunogenicity between MuV-365 and PL-KUM strain antisera in one dose against two kinds of A genotypes (MuV-365 and wild A). There was no significant difference in cross-protection between the antisera in one dose of the two strains tested against the G and F genotypes (Figure 4A). There was no significant difference in immunogenicity between MuV-365 and S79 strain antisera in one dose tested against four kinds of A genotypes (MuV-365, S79, PL-KUM, and wild A). There was no significant difference in cross-protection between the antisera in one dose of the two strains against the G and F genotypes (Figure 4B).

The immune protection of the MuV-365 strain antiserum given in two doses against the S79 strain was significantly higher than that of the PL-KUM strain. There was no significant difference in immunogenicity between the MuV-365 and PL-KUM strain antisera given in two doses against three other kinds of A genotype (MuV-365, PL-KUM, and wild A), and there was no significant difference in cross-protection between the antisera given in two doses of the two strains against the G and F genotypes (Figure 4C).

The immune protection of the MuV-365 strain antiserum given in two doses against the MuV-365 and PL-KUM strains was significantly higher than that of the S79 strain. There was no significant difference in immunogenicity between the MuV-365 and S79 strain antisera given in two doses against two other kinds of A genotypes (wild A and S79), and there was no significant difference in cross-protection between the antisera given in two doses of the two strains against the G and F genotypes (Figure 4D).

**Figure 5:**
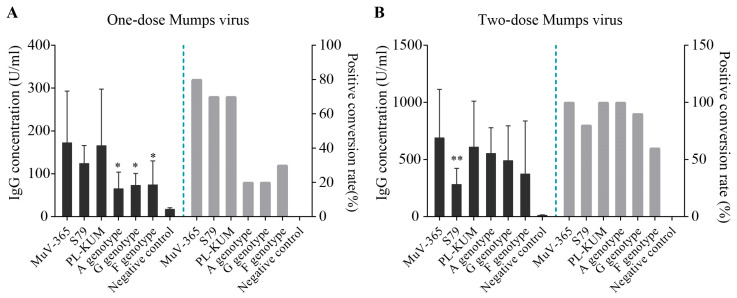
Mumps-specific IgG concentrations. The concentration of mumps virus anti-serum mumps-specific IgG in one dose of MuV-365, S79, PL-KUM, wild A, G, and F strains, respectively (**A**). The concentration of mumps virus anti-serum mumps-specific IgG in two doses of six strains, respectively (**B**). Data are presented as the mean ± SD, *n* = 10. * *p* < 0.05 and ** *p* < 0.01 by unpaired *t*-test.

The concentration of mumps virus antiserum, mumps-specific IgG, in one dose of MuV-365, S79, PL-KUM, wild A, G, and F strains was 173 U/mL, 125 U/mL, 166 U/mL, 66 U/mL,74 U/mL, and 75 U/mL (Figure 5A), respectively, with positive conversion percentages of 80%,70% 70%, 20%, 20%, and 30% (Figure 5A), respectively. The concentration of mumps-specific IgG antiserum of the MuV-365 strain was significantly higher than that of the A, G, and F strains (Figure 5A). The concentration of mumps-specific IgG antiserum in two doses of MuV-365, S79, PL-KUM, wild A, G, and F strains was 692 U/mL, 284 U/mL, 611 U/mL, 556 U/mL, 493 U/mL, and 376 U/mL (Figure 5B), respectively, with positive conversion percentages of 100%, 80%, 100%, 100%, 90%, and 60% (Figure 5B). The concentration of mumps-specific IgG antiserum of the MuV-365 strain was significantly higher than that of the S79 strain (*p* < 0.01), but there was no difference between MuV-365 and other strains (Figure 5B). Furthermore, there was no difference between the MuV-365 strain and the A, G, and F strains (Figure 5B).

#### 3.4.2. Preclinical Immunogenicity Evaluation of the MMR Vaccine Containing the MuV-365 Strain in Rhesus Monkeys

**Figure 6:**
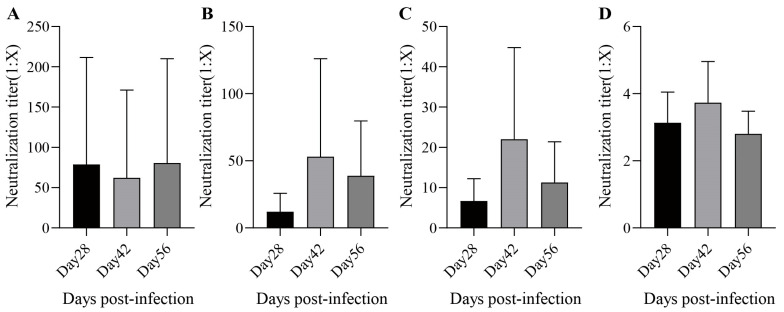
The neutralization antibody titers. The neutralization antibody titers of measles (**A**); mumps (**B**) for A genotype and (**C**) for F genotype; and rubella (**D**) induced by MMR in Rhesus monkeys. Data are presented as the mean ± SD, *n* = 15.

During the rhesus monkey experiments, no deaths and no abnormal clinical symptoms were observed. Under the conditions of this experiment, one dose of MMR was given to rhesus monkeys by subcutaneous injection. The antiserum geometric mean titer (GMT) at 28 days post-infection against measles, mumps (genotype A/F), and rubella virus were 22.9 (Figure 6A), 7.1/5.3 (Figure 6B,C), and 3.0 (Figure 6D), respectively, and the seropositive conversion percentages were 80%, 87%/100%, and 87%, respectively. The antiserum GMT at 42 days post-infection against each virus was 21.4 (Figure 6A), 25.8/15.3 (Figure 6B,C), and 3.5 (Figure 6D), respectively, and the seropositive conversion percentages were 100%, 100%/100%, and 93%, respectively. The antiserum GMTs at 56 days post-infection against each virus were 35.9 (Figure 6A), 22.6/8.0 (Figure 6B,C), and 2.7 (Figure 6D), respectively, and the seropositive conversion percentages were 100%, 100%/100%, and 93%, respectively. In summary, the MMR vaccine had good immunogenicity against measles and rubella after the administration of one dose, while immunogenicity against mumps improved after two doses.

## 4. Discussion

Herein, we have developed a novel MuV vaccine candidate, MuV-365, without amino acid sense variation, as compared to RIT4385. The novel vaccine exhibited preclinically adequate safety and immunogenicity, and it had adequate neutralization activity in mice and monkeys. However, the immunogenicity of these clinical trials is still ongoing.

Despite the high vaccination rate of the mumps vaccine, mumps outbreaks still occur from time to time due to the combination of waning immunity and antigenic mismatch [15]. Developing more effective mumps vaccines based on the characteristics of mumps virus genotype epidemics in different regions is expected to play a better role in immune protection [15]. Although the results of neutralizing-antibody experiments in mice given two doses showed no significant difference in the immune protection of MuV-365 against A, F, and G genotype mumps strains, considering the varying prevalence of different genotypes of mumps strains in different regions, we will still focus on the differences in the protective effects of MMR vaccine containing the MuV-365 strain against different genotypes of mumps strains in subsequent clinical trials to provide data reference for the application of MMR vaccine containing the MuV-365 strain in different regions.

Crucially, neutralizing-antibody levels are essential for vaccine-mediated protection and appear influenced by factors including viral strain antigenic variation, number of vaccine doses received, and inherent limitations of mumps immunity. Our results revealed that one dose of the MMR vaccine generated low mumps neutralization antibody titers, suggesting potentially insufficient protection. However, two doses of the MMR vaccine significantly enhanced efficacy. These results indicate that our developed MMR vaccine requires two immunizations, like the existing MMR vaccine. While MuV-365 induced notably higher neutralizing-antibody titers and mumps-specific IgG concentrations compared to other A genotype strains, such as S79 and PL-KUM, cross-neutralization assays against wild-type G and F genotypes showed no significant difference between MuV-365, S79, and PL-KUM. The universal immune protection of MuV-365 against multiple genotypes of mumps strains can provide good guarantees for its application in the real world, but further confirmation of the advantages of MuV-365 compared to existing vaccines is needed in future clinical studies.

Our analysis of post-vaccination sera also revealed a weak correlation between mumps-specific IgG levels and neutralization titers, aligning with earlier findings [12,14,16]. For MuV, the vaccine-induced antibodies primarily targeted the HN protein and the nucleoprotein, the latter being a non-neutralizing antigen [17]. Crucially, neutralizing antibodies specific to the HN protein constituted only a fraction of the total mumps-specific IgG detected by ELISA in these sera. Consequently, we recommend the MuV cross-neutralization test as the preferred method for evaluating the effectiveness of mumps-containing vaccines.

In the future, our studies will focus on the development of new stabilizer formulations without human blood albumin and gelatin, which caused anaphylaxis symptoms in systemic anaphylaxis experimental guinea pigs.

## 5. Conclusions

In this study, we developed an attenuated mumps vaccine strain that stimulated strong immune responses in BALB/c mice and monkeys. Considering the levels of neutralizing antibodies, IgG concentrations, and the results of toxicity tests, these results highlight the safety, immunogenicity, and efficacy of mumps vaccine candidates using MuV-365.

## 6. Patents

The relevant research results were applied to an invention patent request in China (ZL202311176692.0).

## Figures and Tables

**Table 1:** Dosage and volume for each group of guinea pigs.

Group		Sensitization Day 1, Day 3, and Day 5		Stimulation Day 19 and Day 26	
		Dosage (Dose/Guinea Pig)	Volume (mL/Guinea Pig)	Dosage (Dose/Guinea Pig)	Volume (mL/Guinea Pig)
1	Negative control	0	0.5	0	1
2	Positive control	15 mg/guinea pig	0.5	30 mg/guinea pig	1
3	Low-dose	0.1	0.05	0.2	0.1
4	High-dose	1	0.5	2	1
5	Human blood albumin and gelatin-free control	1	0.5	2	1

**Table 2:** Clinical observations of animals in each group after day 19 foot vein stimulation.

Group	Clinical Observations
Negative control	No anaphylaxis symptoms were found in the three animals, and the anaphylaxis test was negative.
Positive control	Three animals showed different degrees of allergic reaction symptoms, such as hair erection, nose scratching, and gait instability, and the allergic reaction was weakly positive to strongly positive.
Low-dose	Eight animals showed different degrees of anaphylaxis symptoms, such as restlessness, hair erection, nose scratching, cough, urination, defecation, dyspnea, gait instability, convulsion, tidal breathing, and yellow vomit, among which five animals died. One animal showed no anaphylaxis symptoms, and the anaphylaxis was negative to extremely strongly positive.
High-dose	Nine animals showed symptoms of anaphylaxis to varying degrees, such as restlessness, hair erection, trembling, nose scratching, cough, shortness of breath, urination, defecation, dyspnea, purpura, gait instability, wheezing, cramping, and tidal breathing, among which three animals died, with the anaphylaxis weakly positive to very strongly positive.
Human blood albumin and gelatin-free control	No anaphylaxis symptom was found in three animals, and the anaphylaxis was negative.

**Table 3:** Clinical observations of animals in each group after day 26 foot vein stimulation.

Group	Clinical Observation
Negative control	None of the six animals showed any anaphylaxis symptoms, and the anaphylaxis tests were negative.
Positive control	Six animals had different degrees of anaphylaxis symptoms, such as nose scratching, sneezing, coughing, shortness of breath, urination, dyspnea, gait instability, and convulsion, among which two animals died, and the anaphylaxis was positive to extremely positive for anaphylaxis.
Human blood albumin and gelatin-free control	None of the six animals showed anaphylaxis symptoms, and the anaphylaxis test was negative.

## Data Availability

The data that support the findings of this study are available from the corresponding author upon reasonable request.

## Abbreviations

The following abbreviations are used in this manuscript:

MuV	Mumps virus
MMR	Measles–mumps–rubella
MOI	Multiplicity of infection
SH	Small hydrophobic
